# Short-term pulmonary infiltrate with eosinophilia caused by asthma: a phenotype of severe, eosinophilic asthma? Five cases and a review of the literature

**DOI:** 10.1186/s13223-019-0358-x

**Published:** 2019-08-23

**Authors:** Min Song, Shan Cai, Hong Luo, Yi Jiang, Min Yang, Yan Zhang, Hong Peng, Ping Chen

**Affiliations:** 1 Department of Pulmonary and Critical Care Medicine, The Second Xiangya Hospital, Central South University, No. 139 Renmin Road, Changsha, 410011 Hunan China; 2 The Respiratory Disease Research Institute of Hunan Province, The Respiratory Disease Diagnosis and Treatment Center of Hunan Province, No. 139 Renmin Road, Changsha, 410011 Hunan China; 3 Department of Pathology, The Second Xiangya Hospital, Central South University, No. 139 Renmin Road, Changsha, 410011 Hunan China

**Keywords:** Asthma, Eosinophilic pneumonia, Short-term, Intervention

## Abstract

**Background:**

Asthma is often accompanied by peripheral eosinophilia and eosinophilic airway inflammation. This article explores the relationship between asthma and short-term pulmonary infiltrate with eosinophilia, which results from irregular asthma treatment.

**Case presentation:**

We report five unique cases of asthma-induced short-term eosinophilic pneumonia encountered at our pulmonary and critical care centre in Hunan, China, from January 1, 2014, to August 31, 2018. The 5 asthma patients were women with persistent dyspnoea symptoms, an increased peripheral eosinophil count and a high level of exhaled nitric oxide (FeNO). Chest CT revealed multiple infiltrates and ground-glass opacities in both lung fields in all 5 patients. Four of the 5 patients had increased eosinophils in bronchoalveolar lavage (BAL). Three were positive for reversibility in lung function testing, and two had eosinophil infiltration as revealed by lung biopsy. No antibiotic treatment was given, and after a short period of glucocorticoid therapy and inhaled corticosteroid plus long-acting β2-agonist (ICS + LABA) treatment, the symptoms of all of the patients disappeared. In addition, their blood eosinophils returned to normal, and their lung lesions were quickly absorbed and improved.

**Conclusion:**

These cases show a unique association between short-term eosinophilic pneumonia and asthma. The occurrence of eosinophilic pneumonia can prove fatal during a serious asthma attack. Additionally, the presence of peripheral eosinophilia with lung infiltrates poses a diagnostic challenge for clinicians by creating suspicion of pulmonary infiltrate with eosinophilia when present in asthmatic patients.

## Background

Asthma can be associated with mild peripheral eosinophilia, and peripheral blood eosinophilia may be transient, episodic, or persistent [1–3]. Severe asthma is a heterogeneous condition consisting of phenotypes such as eosinophilic asthma [4]. Asthma can be subdivided into eosinophilic or non-eosinophilic phenotypes based on the inflammatory cellular patterns observed in the sputum, blood, and airway tissue compartments. An endotype is the mechanism that drives a subphenotype, including those associated with eosinophilia, such as early-onset allergic asthma with and without obesity, aspirin-sensitive asthma and late-onset eosinophilic asthma, allergic bronchopulmonary mycosis, and exercise-induced asthma [5, 6].

Pulmonary eosinophilia comprises a wide-ranging and heterogeneous group of diseases defined by eosinophilia in pulmonary infiltrates (bronchoalveolar lavage fluid) or in tissue (lung biopsy specimens) and is often accompanied by increased peripheral blood eosinophilia [7–9], including asthma, chronic urticaria, chronic eosinophilic pneumonia (CEP), and hyper-eosinophilic syndrome [10–12]. CEP is a severe disease of unknown cause that has a high recurrence rate [13]. Asthma can precede CEP by several years, and many patients with idiopathic chronic eosinophilic pneumonia (ICEP) develop severe asthma, which, together with relapses, often necessitates prolonged systemic corticosteroid treatment. ICEP may occur in some patients as a rare complication of asthma [14]. The association of ICEP and asthma may be regarded as logical and even expected because both conditions are associated with eosinophilic infiltration of the respiratory tract.

Previous studies have suggested that bronchial asthma and eosinophilic pneumonia are both characterized by eosinophilic infiltration of the lungs, although the sites of eosinophilic infiltration differ. These differences might be caused by heterogeneity in eosinophils [15]. Here, we present 5 patients with asthma accompanied by short-term eosinophilic granulocyte infiltration in the lungs, and the infiltrations observed during treatment for asthma were later clearly fully absorbed.

Unlike the previously reported status of CEP or ICEP with asthma, symptoms and pulmonary lesions of our patients were significantly absorbed after immediate and regular treatment for asthma, inhalation of inhaled corticosteroid and long-acting β2-agonist (ICS/LABA) compound preparation, and there was no recurrence in any patient over more than 1 year of follow-up. Eosinophil ratios in bronchoalveolar lavage (BAL) did not appear at more than 30% among all 5 patients, and the onset of pulmonary symptoms was very short, observations that were different from the common clinical features in CEP or ICEP patients. Regular physical examination also showed the counts and ratios of peripheral blood eosinophils of these patients were normal within 1 year before and after the onset of the disease. The short times of elevated eosinophil counts or ratios in peripheral blood and lung infiltration at the onset of the disease were closely related to the concurrent asthma attack. Therefore, we believe that these may be short-term pulmonary eosinophilic infiltrations induced by asthma itself, representing a clinical subtype of severe eosinophilic asthma.

## Case presentations

### Case 1 (Fig. 1)

**Fig. 1:**
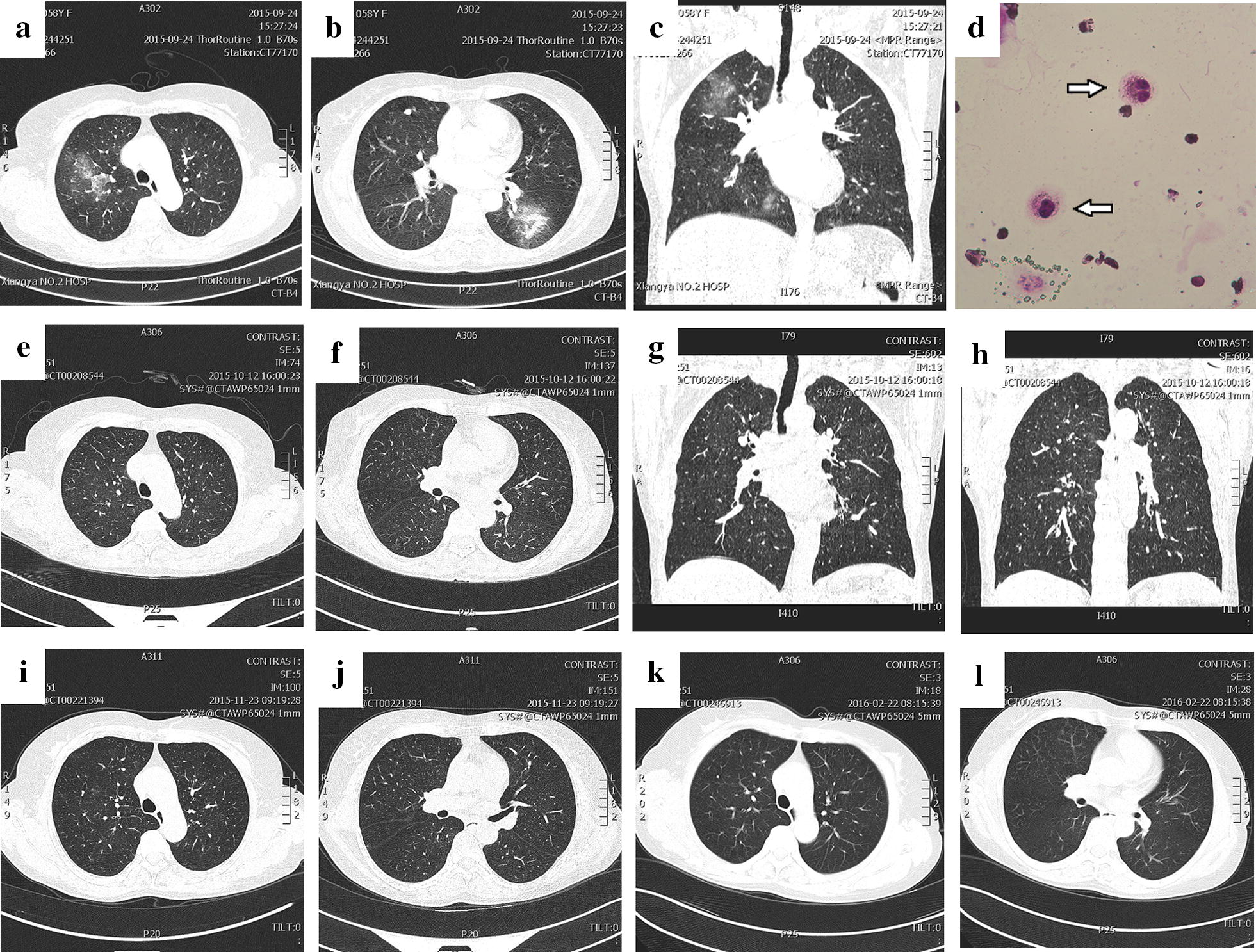
Chest CT images and eosinophils in BAL. **a**–**c** CT scans showed multiple infiltrates in the lower left lung and ground-glass opacities in the subpleural regions of both upper lung fields; **d** eosinophils were observed in BAL (arrow); **e**–**h** double-lung disease was largely absorbed after 2 weeks; **i, j** pulmonary CT scan indicated improvement and resolution of the lesions 2 months later; **k, l** CT scan after 5 months

A 58-year-old woman was referred to our hospital on October 2, 2015, with a 20+-day history of dyspnoea with an increased peripheral eosinophil count (23.9%; 1.17 × 10^9^/L). Physical examination revealed fine crackles over the right lower lung field, a non‑productive cough, and no fever. Blood gas analysis (unoxygenated) results were pH 7.43, PCO_2_ 41 mmHg, PO_2_ 60 mmHg, and HCO_3_—27.2 mmol/L. Chest CT revealed infiltrates in the lower left lung and ground-glass opacities in the subpleural regions of both upper lung fields (Fig. 1a–c). BAL showed 14% eosinophils (244 × 10^5^ mL) (Fig. 1d). Soon after the patient’s symptoms improved significantly, she was treated using methyl prednisone 40 mg for 5 days and inhalation of 160 µg of budesonide and formoterol fumarate powder twice daily. During treatment, the patient had no obvious symptoms, and the chest CT was reviewed after 2 weeks (Fig. 1e–h); a peripheral eosinophil count was also determined (4.0%; 0.17 × 10^9^/L). The two tests of lung function performed before and after treatment are shown in Table 1. Chest CTs were reviewed after 2 and 5 months (Fig. 1i–l) along with a normal peripheral eosinophil count. The patient was a non‑smoker, and she also denied any history of allergic conditions. Blood routine examination in follow-up after 7 months later on April 11, 2016 showed no abnormalities of the eosinophil count and ratio (3.9%; 0.35 × 10^9^/L), with normal frontal and lateral chest radiographs.

**Table 1:** Clinical features of patients with mixed asthma and short-term pulmonary infiltrate with eosinophilia

Subject demographic	Case 1	Case 2	Case 3	Case 4	Case 5
Age (years)	58	29	45	28	57
Sex	Female	Female	Female	Female	Female
Asthma (history)	10 years	2 years	5 months	1 year	4 months
Lung function FVC (L/%)	1.97 (87.7%)	1.83 (53.6%)	3.53 (137%)	2.55 (70%)	2.55 (70%)
FEV_1_ (L/%)	1.17 (62.6%)	1.19 (40.1%)	2.59 (121%)	1.68 (54%)	1.37 (68%)
FEV_1_/FVC (%)	59.52%	65.04%	73%	65%	53.54%
PEF (L/%)	2.80 (51.9%)	3.28 (48.5%)	6.68 (133%)	4.66 (71%)	2.14
Reversibility test	Positive	Positive	Negative	Negative	Positive
FeNO (ppb)	42	40	35	47	40
HRCT findings	Multiple infiltrates in lower left lung and ground-glass opacities in the subpleural regions of both upper lung fields	Multiple ground-glass opacities in the regions of both lung fields	Multiple small nodules in the upper lung	Multiple infiltrates in upper left lung, lower right lung and ground-glass opacities in the subpleural regions of both upper lung fields	Multiple infiltrates in upper left lung, lower right lung and ground-glass opacities in the subpleural regions of both upper lung fields
Blood Eos (*10 ~ 9/L, %)	1.17 (23.9%)	1.86 (18.7%)	0.64 (17.6%)	1.57 (29.3%)	1.31 (18.9%)
Serum IgE (ng/ml)	3715	7951	5697	3672	6120
Bronchial lavage eosinophils (%)	14%	16%	27%	28%	Not performed
Treatment					
Methylprednisolone 40 mg ivgtt 5 days + Budesonide and Formotero (160 µg, inhalation, Bid)	Done	Done	Done	Done	Done
Blood Eos (*10 ~ 9/l, %)	0.17 (4.0%)	0.65 (12.2%)	0.12 (1.5%)	0.43 (0.2%)	0.10 (0.1%)
Lung function FVC (L/%)	2.09 (93.04%)	3.08 (92.4%)	Not performed	Not performed	2.87
FEV_1_ (L/%)	1.32 (67.7%)	1.87 (64.3%)			1.90 (83.1%)
FEV_1_/FVC (%)	63.00%	60.57%			68.76%
PEF (L/%)	3.45 (71.9%)	4.6 (68.8%)			6.09
Reversibility test	Positive	Positive			Positive
FeNO (ppb)	14	14			18
HRCT findings	Apparent absorption	Apparent absorption	Apparent absorption	Apparent absorption	Apparent absorption

### Case 2 (Fig. 2)

**Fig. 2:**
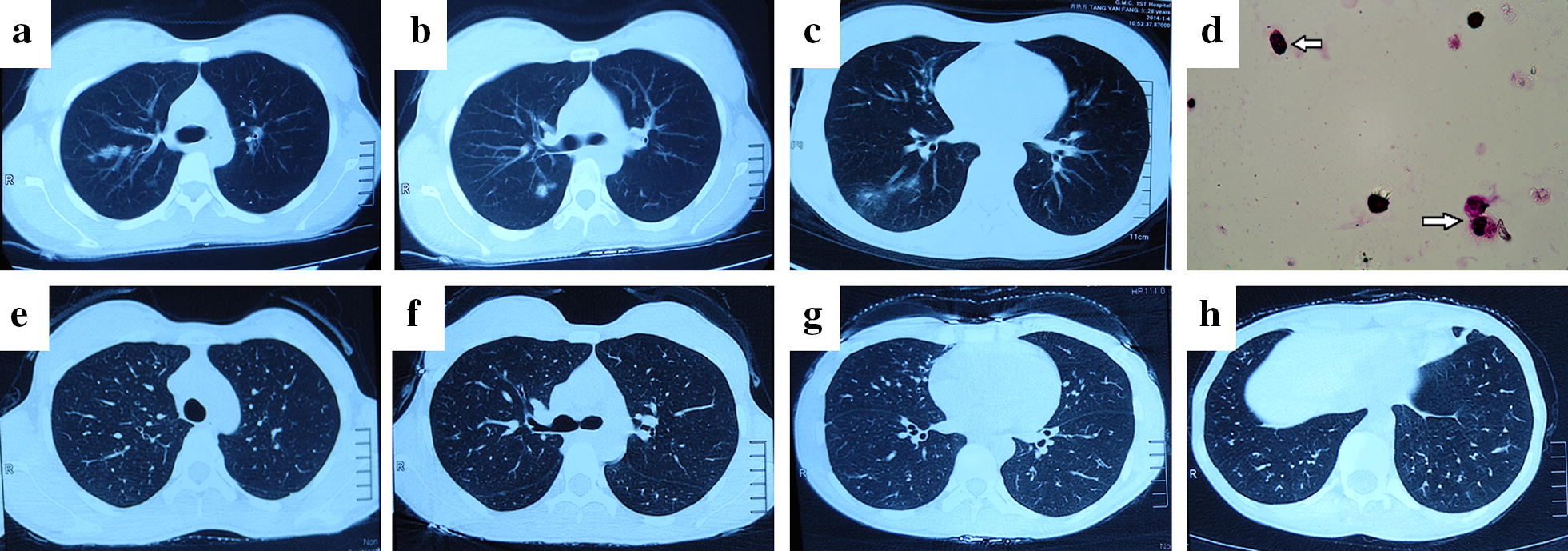
Chest CT images and eosinophils in BAL. **a**–**c** CT scans showed multiple ground-glass opacities in the regions of both lung fields; **d** eosinophils in BAL (arrow); **e**–**h** double-lung disease was largely absorbed after treatment

A 29-year-old woman had a history of repeated cough, expectoration, wheezing, and dyspnoea for 2 years. Her peripheral blood tests at this visit revealed an increased peripheral eosinophil count (18.7%, 1.86 × 10^9^/L). Her routine blood tests during vaginal delivery 6 months ago showed no abnormally elevated eosinophil count or ratio. Her blood gas analysis (unoxygenated) results were pH 7.45, PCO_2_ 34 mmHg, PO_2_ 63 mmHg, and HCO_3_—23.6 mmol/L. Physical examination revealed rough wheezing over both sides of the lung field. Bronchoalveolar lavage (BAL) showed 16% eosinophils. Chest CT images revealed multiple ground-glass opacities in the regions of both lung fields (Fig. 2a–c). BAL showed 17% eosinophils (260 × 10^5^ mL) (Fig. 2d). After receiving an intravenous drip of 40 mg of methylprednisolone for 5 days, the patient’s symptoms improved significantly, and the treatment was followed with inhalation of 160 µg of budesonide and formoterol fumarate powder twice daily. The patient’s chest CT images were reviewed after 1 month (Fig. 2e–h). The changes in lung function are shown in Table 1. The patient had a history of allergic reactions to “penicillin” and “levofloxacin”. She was treated for asthma with regular inhalation of 160 µg of budesonide and formoterol fumarate powder twice daily, and there were no abnormal blood routine or chest imaging findings at follow-up 1 year later.

### Case 3 (Fig. 3)

**Fig. 3:**
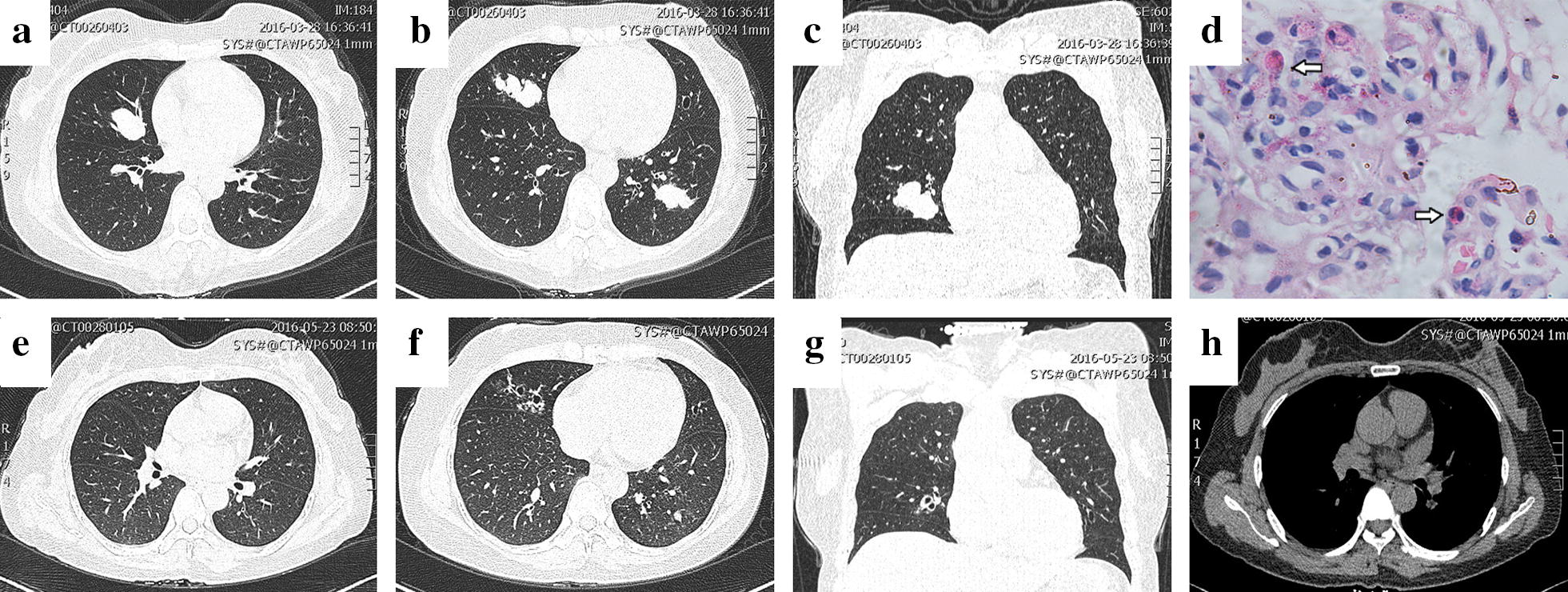
Chest CT images and eosinophils in biopsy specimens. **a–c** Chest CT revealed a number of small nodules in the upper lung and densities in the right middle lung and lower left lung; **d** pathological biopsy of the lower left pulmonary mucosa revealed that more eosinophils (arrow) and lymphocytes had infiltrated the local interstitium, and chronic inflammation was observed in the mucosa; **e–h** chest CT was re-performed after 1 month

A 45-year-old woman with a history of repeated chest tightness, dyspnoea, and back pain for more than 5 months had an increased peripheral eosinophil count (17.6%; 0.64 × 10^9^/L). Physical examination revealed rough wheezing over both sides of the lung field. Chest CT revealed densities in the right middle lung and lower left lung and a number of small nodules in the upper lung (Fig. 3a–c). Under a bronchoscope, there was a purulent discharge in the bronchial segment of the outer base of the lower left lobe. A pathological biopsy of the lower left pulmonary mucosa showed that the local interstitium exhibited more eosinophil and lymphocyte infiltration and chronic inflammation of the mucosa (Fig. 3d). Lung function test results are shown in Table 1. After receiving an intravenous drip of 40 mg of methylprednisolone for 5 days, followed by inhalation of 160 µg of budesonide and formoterol fumarate powder twice daily, the patient’s symptoms improved significantly. Her chest CT was reviewed after 2 months (Fig. 3e–h) and showed a normal peripheral eosinophil count (1.5%; 0.12 × 10^9^/L). Blood routine examination in follow-up after 2 years on March 7, 2018, showed a normal eosinophil count and ratio (7.7%; 0.33 × 10^9^/L).

### Case 4 (Fig. 4)

**Fig. 4:**
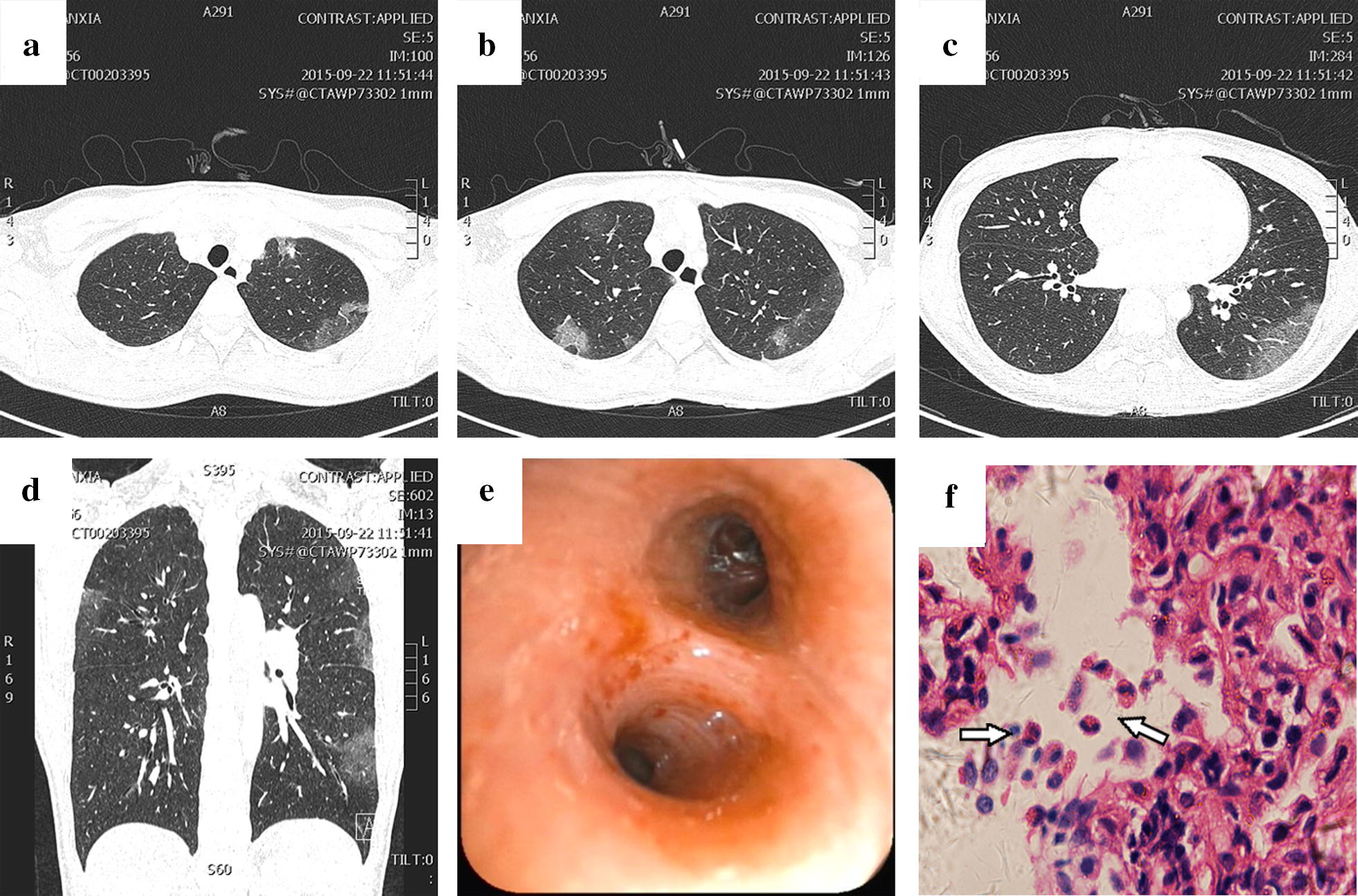
Chest CT images, bronchoscopy and histopathological examination. **a**–**d** Chest CT showed multiple infiltrates in the upper left lung and lower right lung and ground-glass opacities in the subpleural regions of both upper lung fields; **e** the mucosa of the left lower lobe bronchus was found to exhibit hyperaemia and swelling by bronchoscopy; **f** (haematoxylin–eosin staining, ×40) a pathological biopsy of the lower left lung base segment showed that a small number of eosinophils (arrow) had infiltrated the lung

A 28-year-old woman with a history of repeated cough, dyspnoea and wheezing for more than 1 year and blood in the sputum for 5 days presented with an increased peripheral eosinophil count (29.30%; 1.57 × 10^9^/L). Physical examination revealed thick breath sounds over both sides of the lung field. Blood gas analysis (unoxygenated): pH 7.42, PCO_2_ 39 mmHg, PO_2_ 66 mmHg, and HCO_3_—25.3 mmol/L. Chest CT revealed multiple laminated glass shadows in the lung (Fig. 4a–d). A pathological biopsy of the lower left lung base segment showed that a small number of eosinophils had infiltrated the lung (Fig. 4f). Lung function test results are shown in Table 1. The patient had a history of allergies to dust mites and milk. After an intravenous drip of 40 mg of methylprednisolone for 5 days, followed with regular inhalation of ICS/LABA compound preparation (160 µg of budesonide and formoterol fumarate), she had no recurrence of these symptoms. She had reduced the number of inhalations a year earlier under the guidance of an asthma specialist and had no other attacks for more than 1 year.

### Case 5 (Fig. 5)

**Fig. 5:**
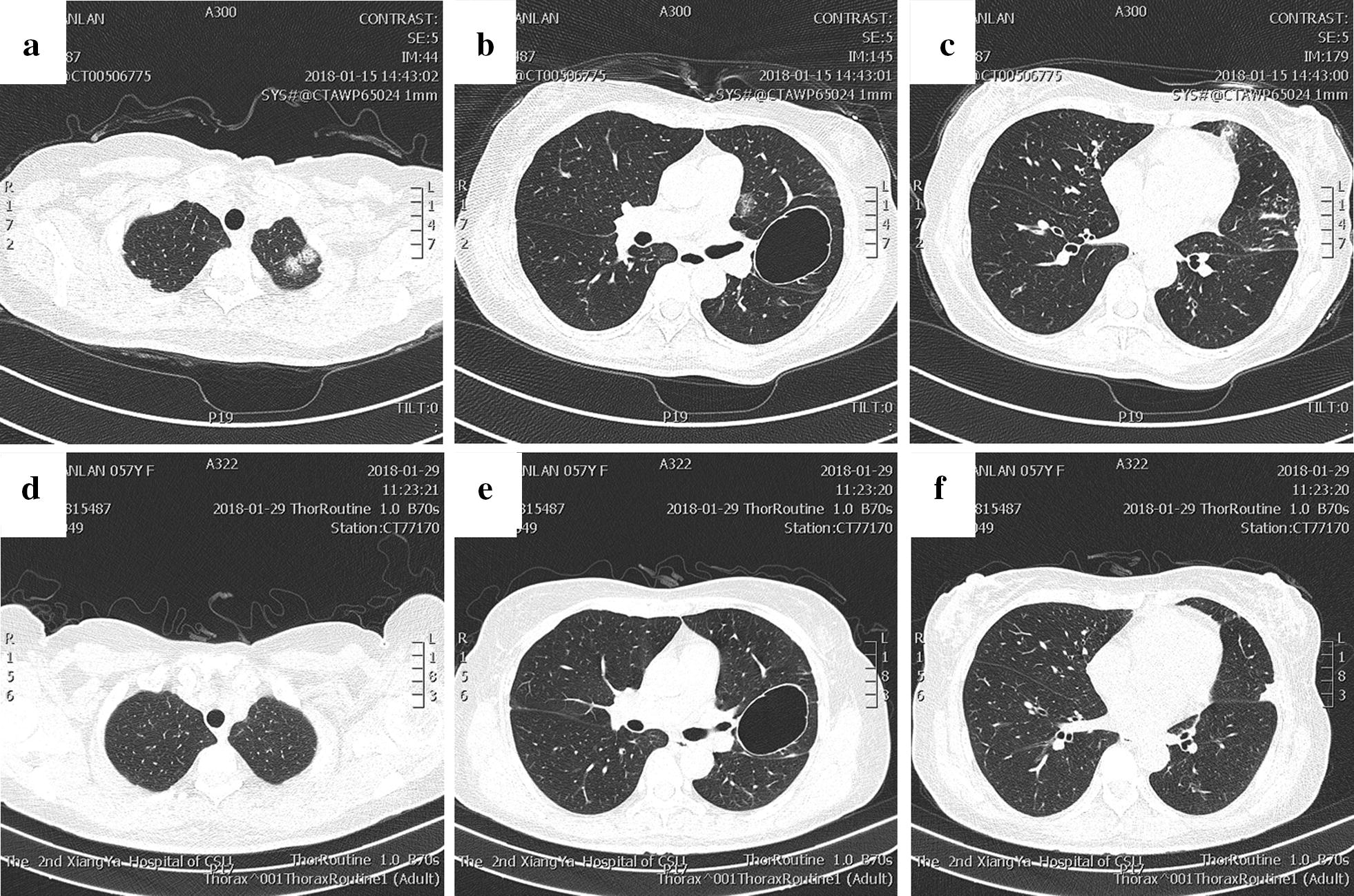
Chest CT images. **a**–**c** Chest CT showed multiple infiltrates in the upper left lung and lower right lung and ground-glass opacities in the subpleural regions of both upper lung fields; **d**–**f** the lesion in the lower right lobe was partially absorbed

A 57-year-old woman with a history of repeated coughing and phlegm for 4 months aggravated with dyspnoea for 12 days presented with an increased peripheral eosinophil count (18.90%; 1.31 × 10^9^/L). However, on November 21, 2016, the count and ratio of eosinophils were normal in a routine examination of peripheral blood (3.7%; 0.2 × 10^9^/L). Physical examination revealed thick breath sounds over both sides of the lung field. Blood gas analysis (unoxygenated) results were pH 7.38, PCO_2_ 39.8 mmHg, PO_2_ 54.4 mmHg, and HCO_3_—22.9 mmol/L. Chest CT revealed multiple laminated glass shadows in the left lung (Fig. 5a–c). She was diagnosed with severe asthma with short-term intravenous hormone therapy. The lung disease was largely absorbed after she used methylprednisolone 40 mg for 5 days (Fig. 5d–f), and she had a normal peripheral eosinophil count (0.1%; 0.10 × 10^9^/L). Her lung function test results are shown in Table 1. She had no history of allergies. During her follow-up on February 20, 2019, she had no recurrence of symptoms with inhaled medication of ICS/LABA compound preparation (160 µg of budesonide and formoterol fumarate), and her blood tests showed a normal eosinophil count and ratio (6.8%; 0.35 × 10^9^/L).

## Discussion and conclusions

In the above five cases, we observed similar phenomena. Infiltrated shadows were observed in asthma patients with elevated peripheral blood eosinophils. Invasive bronchoscopy to obtain pathological examination results for BAL and lung mucosa tissue revealed that the BAL eosinophil:granulocyte ratio was increased. Disease screening also showed mucosa and acidophilic granulocyte infiltration in the lung tissue. Eosinophilic lung disease exhibits diverse pathological characteristics. This condition may be idiopathic or caused by various factors, including drugs, infection, allergies, hazardous materials, smoking and vasculitis. Some eosinophilic disorders, such as allergic bronchopulmonary aspergillosis and eosinophilic granulomatosis with polyangiitis, can involve both parenchymal and airway structures [16, 17]. Asthma is characterized by airway inflammation rich in eosinophils, and airway eosinophilia is associated with exacerbations and has been suggested to play a role in airway remodelling. However, severe eosinophilic asthma combined with pneumonia is rare [18], and cases of eosinophilic pneumonia caused by asthma itself have not been reported. Peripheral eosinophilia is often observed when tissue levels are elevated, but this is not as reliable a marker as tissue biopsy. In the above five cases, cytology from BAL and histology from transbronchial biopsies showed pulmonary eosinophil infiltrates [19].

CEP is a disease of unknown cause. The hallmark of CEP is eosinophil accumulation in the lungs, and asthma is not a prerequisite for the development of CEP [20]. Many patients with ICEP develop severe asthma, which, together with relapses, often necessitates prolonged systemic corticosteroid treatment. ICEP may occur in some patients as a rare complication of asthma, although it is seldom mentioned in reviews and textbooks on asthma. Furthermore, when present in patients with ICEP, asthma is relatively severe and worsens after the diagnosis of ICEP. In addition, the presence of asthma at the time of a diagnosis of ICEP has been associated with fewer relapses of ICEP, possibly because these patients have a higher frequency of long-term inhaled corticosteroid (ICS) use in asthma [21]. The association between ICEP and asthma may be logical and even expected because both conditions are associated with eosinophilic infiltration of the respiratory tract. Approximately half of ICEP patients (51.6%) had a previous and often prolonged history of asthma [14]. Exploring the links between asthma and ICEP could increase the understanding of the mechanisms underlying hyper-eosinophilic lung diseases [22]. ICEP is twice as common in women as in men. One-third to one-half of all ICEP patients have a history of asthma [23]. A study of 62 ICEP patients performed in France showed that 59 (95.2%) presented severe eosinophilia (eosinophil count greater than 1000 cells/mm^3^). The mean percentage of eosinophils was 30%, and BAL analysis revealed eosinophilia (greater than 25% eosinophils in all cases and greater than 40% eosinophils in 80% of the cases). The prevalence of smokers was low (6.5%) [13]. However, our cases were obviously different in that all 5 patients exhibited typical symptoms, had a previous medical history of asthma, and had mild to moderately increased peripheral blood of acidophilic granulocytes; after a few days of treatment with intravenous use of methylprednisolone, they quickly returned to normal levels. BAL examination showed that the proportion of eosinophils was less than 30% in 4 of the patients. The eosinophil counts and ratios were normal in all of the patients during regular physical examinations within 6 months to 2 years before or after the onset of the attack visit. Therefore, we wondered whether these patients might have peritoneal eosinophil infiltration of the lungs and whether the increased ratio of peripheral blood eosinophils was caused by asthma itself, as this is a phase of the asthma process.

Another hypothesis was that these signs were early signs and characteristics of combined CEP in patients with asthma. However, in terms of treatment and recurrence, based on the diagnosis of asthma, all 5 patients in this paper were treated with intravenous glucocorticoid therapy for only 5 days, and the inhalation of ICS/LABA was the main treatment. During the follow-up, there was no evidence of pulmonary disease recurrence after receiving regular treatment for asthma. However, in the literature of Marchand et al. [14], the vast majority of CEP patients required prolonged OCST and could relapse extremely easily to within 6 months, even during long-term oral hormone therapy. The difference is that there was no recurrence in the above 5 patients over more than 1 year of follow-up with short-term oral corticosteroid and ICS/LABA inhalation. In any case, these patients require longer follow-up to monitor and subsequently assess asthma control levels, peripheral blood/BAL eosinophils, and chest CT status. Our patients were successfully treated with short-term oral corticosteroid and inhalation medication in the early phase, which prevented complications. All 5 of our patients were female, and the mainstay treatment was a few days of systemic corticosteroids and long-term inhaled bronchial diastolic agents. We cannot predict what types of diseases these patients will suffer from in the future, and we cannot rule out the possibility that CEP may occur in these patients in the future. We may have observed the early signs of overlap between these two diseases, and a long follow-up period of clinical observation is therefore important. Moreover, whether this is a phenotype of severe asthma also needs to be studied with long-term follow-up and regular review.

Some people with severe asthma have eosinophilic asthma, and uncontrolled eosinophilic airway inflammation is associated with a reduced response to glucocorticoids and an increased risk of severe exacerbations [24]. Diagnosing eosinophilic asthma is important because it is based on measurements of sputum eosinophils, blood eosinophils, FeNO, serum IgE and periostin, which are used as surrogates [25]. All 5 of our patients had increased blood eosinophils, increased FeNO and serum IgE levels, and pulmonary eosinophil infiltrates. However, the relationships between these factors and the eosinophil ratio and eosinophilic pneumonia in BAL are not very clear. The alveolar lavage test is time consuming and requires specific technical expertise; additionally, the condition of patients with severe asthma makes this invasive test risky with unpredictable complications. Late-onset asthma was associated with the highest numbers of lung eosinophils (P < 0.007), while early-onset severe asthma was associated with a lymphocytic/mast cell inflammatory process [26]. An early-onset atopic type, obesity, and non-eosinophilic were common to both asthma populations, but a marked discordance between symptom expression and eosinophilic airway inflammation (early-onset symptom-predominant and late-onset inflammation-predominant) was specific to refractory asthma [27]. The 5 patients reported in this article had late-onset asthma, with asthma symptoms and diagnoses occurring after an age of 12 years old. In some cases, asthma-like symptoms occurred at an age of 40 years old or even after an age of 50 years old. The condition of the late-onset eosinophilic asthma patients was severe, and the patient in case 5 was in respiratory failure at the time of onset. The presence of persistent sputum eosinophilia despite extensive antiasthma treatment is not a refractory phenomenon but is still sensitive to high-dose systemic corticosteroids. Patients with severe asthma need additional or alternative anti-inflammatory treatments to combat eosinophilia and its associated poor prognosis [28]. In our 5 patients, FeNO levels were higher than 35 ppb, and the ratio of eosinophils in the peripheral blood was increased. After a short period of glucocorticoid administration in systemic veins, the ratio of eosinophils and the level of eosinophils in the peripheral blood had significantly decreased. Moreover, multiple high-density shadows observed on high-resolution CT of both lungs were also significantly absorbed within 1 week or several weeks on re-examination. These results suggest that in asthmatic patients, pulmonary lesions follow the changes in the ratio of eosinophils in peripheral blood and the levels of FeNO. The lung biopsy results in some patients also indicated that the lungs experienced a short period of eosinophil infiltration during this process, and the response to glucocorticoid therapy was very good. Determining whether these patients should be classified as severe asthma, refractory asthma or eosinophilic asthma will require more clinical cases to be summarized.

In rare cases, cutaneous larva migrans may be complicated by Löffler syndrome. This syndrome is characterized by migratory pulmonary eosinophilic infiltrates and peripheral eosinophilia with malaise, fever, and cough [29–32]. However, the above 5 patients in this study were diagnosed with asthma, and they all had a short period of lung acidophilic granulocyte infiltrations due to a lack of regular anti-asthma treatment. Fortunately, these pathological changes in the patients’ lungs were clearly absorbed after treatment with glucocorticoids, and there was no recurrence of pulmonary lesions following asthma treatment.

The identification of these findings has led to a personalized management approach to this condition consisting of improved diagnostic techniques, which has improved stratification in and more effective treatment of patients. The presence of peripheral eosinophilia with lung infiltrates poses a diagnostic challenge for the clinician. We sometimes had difficulty in making a differential diagnosis between Löffler syndrome and eosinophilic pneumonia caused by asthma. The purpose of this article is to further explore eosinophilic presence, activity, and pathology in the respiratory tract and to discuss current and future treatment options through a detailed literature review.

## Data Availability

This materials has not been published in whole or in part elsewhere and has been obtained with the consent of the Hospital and the Patients. Please contact the author for data requests.

## Abbreviations

ICEP: idiopathic chronic eosinophilic pneumonia
CEP: chronic eosinophilic pneumonia
FeNO: exhaled nitric oxide
BAL: bronchoalveolar lavage
FVC: forced vital capacity
FEV_1_: forced expiratory volume in one second
PEF: peak expiratory flow
ICS: inhaled corticosteroid
LABA: long-acting β2-agonist
